# Programmed Cell Death 10 Mediated CXCL2-CXCR2 Signaling in Regulating Tumor-Associated Microglia/Macrophages Recruitment in Glioblastoma

**DOI:** 10.3389/fimmu.2021.637053

**Published:** 2021-05-24

**Authors:** Quan Zhang, Junwen Wang, Xiaolong Yao, Sisi Wu, Weidong Tian, Chao Gan, Xueyan Wan, Chao You, Feng Hu, Suojun Zhang, Huaqiu Zhang, Kai Zhao, Kai Shu, Ting Lei

**Affiliations:** ^1^ Department of Neurosurgery, Tongji Hospital, Tongji Medical College, Huazhong University of Science and Technology, Wuhan, China; ^2^ Department of Neurosurgery, Beijing Tiantan Hospital, Capital Medical University, Beijing, China; ^3^ Department of Neurosurgery, The Third People’s Hospital of Hubei Province, Wuhan, China; ^4^ Department of Neurosurgery, First Affiliated Hospital of Medical College, Shihezi University, Xinjiang, China

**Keywords:** glioblastoma, CXCR2, CXCl2, SB225002, tumor-associated microglia/macrophages (TAM), programmed cell death 10 (PDCD10)

## Abstract

**Background:**

Programmed cell death 10 (PDCD10) plays a crucial role in regulating tumor phenotyping, especially in glioblastoma (GBM). Glioma-associated microglia/macrophages (GAMs) in tumor pathological microenvironment contribute to GBM progression. We previously found that the infiltration of GAMs was associated with PDCD10 expression in GBM patients. The present study aims to further explore the regulation of PDCD10 on GAMs in GBM.

**Methods:**

Overexpression of *PDCD10* in human- and murine-GBM cells was established by lentiviral transduction. Cell behaviors and polarization of primary microglia, microglia- and macrophage-like cells were investigated through indirect co-culture with GBM cells *in vitro* respectively. The PDCD10-induced release of chemokines was identified by a chemokine protein array. The cross-talk between GBM and microglia as well as macrophages was further studied using selective antagonist SB225002. Finally, an orthotopic homograft mouse model was employed to verify the results of *in vitro* experiments.

**Results:**

Indirect co-culture with *PDCD10-*overexpressed GBM cells promoted proliferation and migration of microglia- and macrophage-like cells, and stimulated pro-tumorigenic polarization of primary microglia, microglia- and macrophage-like cells. *Pdcd10*-upregulated GBM cells triggered a nearly 6-fold increase of CXC motif chemokine ligand 2 (CXCL2) release, which in turn activated CXC chemokine receptor 2 (CXCR2) and downstream Erk1/2 and Akt signaling in primary microglia, microglia- and macrophage-like cells. The blockage of CXCR2 signaling with specific inhibitor (SB225002) abolished microglia- and macrophage-like cell migration induced by *PDCD10*-upregulated GBM cells. Moreover, *Pdcd10*-upregulated GL261 cells promoted GAMs recruitment and tumor growth *in vivo*.

**Conclusion:**

Our study demonstrates that overexpression of *PDCD10* in GBM recruits and activates microglia/macrophages, which in turn promotes tumor progression. CXCL2-CXCR2 signaling mediated by PDCD10 is potentially involved in the crosstalk between GBM cells and GAMs.

## Introduction

Glioblastoma (GBM) is the most common primary malignant tumor in central nervous system, accounting for 57.3% of gliomas with poor prognosis (1). Five-year survival rate is only 6.8% despite the combine application of aggressive surgical resection, chemotherapy and radiation (2). Characterized as a heterogeneous neoplasm, tumor microenvironment (TME) in GBM consists of various types of cells including tumor cells, endothelial cells, tumor-associated microglia/macrophages (TAMs) and numerous soluble factors like cytokines. TAMs, which are also named as glioma- associated microglia/macrophages (GAMs), account for up to 30% of tumor mass in human GBM (3). The interaction between tumor cells and TME modulated tumor progression (4). GAMs are composed of resident microglia and monocyte-derived macrophages (MDMs) which enter the brain through the compromised blood brain barrier under pathologic status (5). Accumulating evidences suggest that TAMs play a crucial role in tumorigenesis and progression (6, 7). The accumulation and activation of GAMs in GBM niche promoted tumor growth and invasion, while depletion of GAMs inhibited tumor growth (8, 9). Novel therapeutic strategies targeting TAMs have achieved a curative effect in various cancers *in vitro* (10, 11). However, these medications did not improve the patient prognosis (7). The infiltration of TAMs in tumors was mediated by numerous chemotactic factors produced by tumor cells and TME cells including C-C motif ligand 2 (CCL2), CX3C chemokine ligand 1 (CX3CL1), stromal cell-derived factor-1 (SDF-1), colony-stimulating factor-1 (CSF-1) and periostin (POSTN) etc. (12). These facts raised our consideration to define a novel therapeutic approach targeting the interaction of tumors and GAMs.

Programmed cell death 10 *(PDCD10)*, originally named TF-1 cell apoptosis related gene 15, is also known as cerebral cavernous malformation 3 (*CCM3*) (13). Mutations of CCM3 resulted in human familial cerebral cavernous malformation. PDCD10 is widely expressed in various types of cells, such as astrocyte, neuron, endothelial cells and tumor cells. PDCD10 is the component of striatin-interacting phosphatase and kinase (STRIPAK) complex, plays an important role in the modulation of germinal center kinases III activity, vascular endothelial-derived growth factor receptor 2 (VEGF-R2) internalization, Golgi assembly and cell polarity (14). Endothelial loss of *PDCD10* promoted cell proliferation, migration, sprouting and tube formation (13). In central nerve system, PDCD10 played a critical role in neuron-glial unit and neo-neuron migration (15, 16). *Pdcd10*-deficiency in gut epithelium augmented CCM formation in a mouse model, indicating the PDCD10 function in a gut-brain axis (17). Altered expression of *PDCD10* had been reported in various tumors including breast cancer, prostate cancer, bladder cancer etc., playing dual function in tumor progression and chemo-therapy resistance (18–20).

The function of PDCD10 in GBM is still unclear. Previous report indicated that loss of *PDCD10* in GBM activated tumor cell behaviors and mediated chemotherapy resistance (21). However, clinical data from The Cancer Genome Atlas (TCGA) shows that *PDCD10* is upregulated in GBM patients, revealing a positive correlation with poor prognosis (**Figure S1**). We focused on PDCD10 function and reported previously a paracrine mechanism triggered by endothelial-PDCD10 (22). Most recently, we found that GAMs infiltration was positively correlated with PDCD10-positive staining in specimens generated from GBM patients, which was consistent with the findings from TIMER database described in **Figure S2**. Taken together, we assumed that PDCD10 in GBM played a role in the recruitment and activation of GAMs. To this end, we performed indirect co-culture experiments and employed an orthotopic homograft mouse model to investigate the underlying mechanism. As a result, we demonstrate overexpression of PDCD10 in GBM recruits and activates microglia/macrophages, which in turn promotes tumor progression. CXC motif chemokine ligand 2/CXC motif chemokine receptor 2 (CXCL2-CXCR2) signaling mediated by PDCD10 is potentially involved in the crosstalk between GBM cells and GAMs.

## Material and Methods

### GBM Patients’ Samples

Thirty-four specimens were obtained from patients who underwent surgical treatments from 2015 to 2018 in Tongji Hospital and histologically confirmed as GBM postoperatively. This study was approved by Ethical Committee of Tongji Hospital, Tongji Medical College, Huazhong University of Science and Technology in accordance with the Helsinki Criteria. Informed consent was obtained from all individual patients included in this study.

### Animals

C57BL/6J mice used for isolation of primary microglia or for orthotopic homograft implantation were purchased from SPF Biotechnology Co. Ltd., Beijing, China. C57BL/6J mice of mixed sex were bred and kept in the animal center of Tongji Medical College, Huazhong University of Science and Technology. All the experiments were performed following the ARRIVE (Animals in research reporting *in vivo* experiments) Guidelines and were approved by the Committee on Animal Research of Tongji Medical College, Huazhong University of Science and Technology.

### Cell Culture and Mouse Primary Microglial Isolation

Murine GBM cell line GL261 (National Cancer Institute, Frederick, USA), microglia-like cell line BV2 and human GBM cell lines U251 (China Center for Type Culture Collection, CCTCC, Wuhan, China), murine monocyte/macrophage cell line RAW264.7 and human GBM cell lines U373 (American Type Culture Collection, ATCC, Manassas, USA) were cultured in Dulbecco’s modified Eagle’s medium (DMEM, Gibco, Carlsbad, USA) supplemented with 10% fetal bovine serum (FBS, Gibco), 200 mM glutamine, 50 units/ml penicillin and 50 μg/ml streptomycin. Human monocyte cell line THP1 (ATCC) were cultured in RPMI1640 (Gibco) with 10% FBS and 200 mM glutamine. To perform following experiments, THP1 was pretreated with phorbol myristate acetate (PMA, Sigma-Aldrich, St. Louis, USA) 100 ng/ml for 2 days, to obtain differentiated macrophage-like cells (THP1-mac).

Mouse primary microglia was isolated from neonatal C57BL/6J mice. Briefly, the forebrains were completely digested by 0.125% trypsin and DNase (Sigma) and centrifuged at 1500 rpm for 15 min. The cell pellet was suspended in culture medium and filtrated with a 40 μm cell filter (Millipore, Darmstadt, Germany). Then, the cell mixture was incubated in a poly-L-lysine pre-coated flask for 14 days. Microglia detached after shaking flask at 220 rpm for 1 h at 37 °C. The supernatant containing microglia was collected and centrifuged (1000 rpm for 5 min). Then, microglia were routinely cultured in DMEM with10% FBS. Iba1 staining was used for verification of isolated primary microglia.

### Human *PDCD10* and Murine *Pdcd10 -*Upregulated GBM Cell Line Generation

The lentiviral vectors for human *PDCD10* (ox*PDCD10*, Cat#29650-13) and murine *Pdcd10* (ox*Pdcd10*, Cat#43748) and corresponding empty vectors (LVCON238 for human species, Ch and LVCON254 for murine, Cm) were purchased from GenePharma, Shanghai, China. The infection of lentiviral-*PDCD10/Pdcd10* and corresponding controls in human (U251 and U373) and murine GBM cell lines (GL261) was respectively performed according to manufacturer’s instructions. After selection by 2 μg/μl puromycin (Sigma), the PDCD10 upregulation was confirmed by RT^2^-PCR and Western Blot.

### Cell Proliferation, Migration and Cell Morphology Study

The conditioned medium (CM) and control medium (C) were prepared from ox*PDCD10*/ox*Pdcd10* and corresponding empty vectors transducted GBM cells respectively. Briefly, the same number of GBM cells was incubated in culture dishes with equal volume of DMEM supplemented with 10% of FBS and 200 mM glutamine for 48 hours. Then, the media were harvested, centrifuged and used for the following experiments. Cell behavior studies were performed by incubation with CM or control medium. For cell proliferation assay, 5.0 x 10^3^ cells were seeded into 96-well-plate with respective media derived from GBM cells. After 72 hours incubation, the cell proliferation was detected by CCK8 reagent (Boster Biological Technology, Pleasanton, USA). To perform scratch assay, 5.0 x 10^5^ cells were seeded in 6-well-plate overnight. A thin stripe was scratched by a pipette tip. Then, cells were incubated with CM or control medium for 24 hours. The migrated area was photographed by a microscope (Olympus Corporation, Tokyo, Japan). For transwell assay, 3 x 10^6^ *PDCD10/Pdcd10*-upregulated- or control-GBM cells with corresponding medium was added into 24-well-plate. Then, 1.0 x 10^5^ cells were suspended and seeded in the inserts of transwell system (8 μm pore, Corning Life Sciences, NY, USA), which was placed in 24-well-plate. After 48 hours incubation, migrated cells were fixed with 4% of formalin and stained with crystal violet. The numbers of migrated cells were counted. The morphology of cells including primary microglia, microglia- and macrophage-like cells was observed through regular microscope. The number of spindle-shaped cells and the average length of cellular processes were measured using Image J software. To determine whether inhibition of CXCR2 signaling suppressed microglia- and macrophage-like cell migration induced by ox*PDCD10*-GBM cells, SB225002 (Cat#S7651, Selleck Chemicals, Houston, USA), a selective CXCR2 antagonist, was used with optimized concentrations. Generally, each *in vitro* experiment was performed for at least 3 times with 3 technical replicates.

### RT^2^-PCR and Western Blot

RNA extraction was performed using Axygen AxyPrep Kit (Corning Life Sciences) according to the manufacture’s protocol. cDNA was synthesized using PrimeScript RT Kit (Takara Bio Inc., Shiga, Japan). The PCR reaction was performed using TB Green kit (Takara Bio Inc.) with standard procedure in CFX Systems (Bio-Rad, Hercules, USA). Sequences of human and mouse primers were shown in **Supplemental Data** (**Table S1**). Relative expression of target genes was quantified using 2^-ddCt^ method, normalized to the reference gene.

Protein extraction and Western blot were carried out as previous description (22). The following antibodies was used: PDCD10, GAPDH (each 1:1000 dilution, Abcam, Cambridge, UK), Iba1 (1:500, Abcam), CXCR2 (1:500, Abclonal, Wuhan, China), p-Erk1/2, Erk1/2, p-Akt and Akt (each 1:1000 dilution, Cell signaling technology, Danvers, USA).

### Protein Array

The protein array was carried out using a mouse chemokines array kit (Cat#: ARY020, R&D Systems, Minneapolis, USA). Two identical membranes precoated with antibodies against 25 different chemokines were incubated with media derived from ox*Pdcd10*- and control-GL261 cells respectively. The following procedure was performed according to the manufacturer’s instructions. The semi-quantification of the dots was analyzed by ImageJ software.

### Immunohistochemical (IHC) and Immunofluorescent (IF) Staining

IHC and IF staining were respectively performed according to the protocols described in our previous publication (22). We did use PBS and related lysis buffer to perfuse blood cells before IHC and IF for GAMs. For IHC-staining, the sections were incubated with primary antibodies as follows: PDCD10 (1:200) and anti-Iba1 (1:200). Images were acquired by an Olympus microscope. To perform double-IF staining, the following primary antibodies mixtures were applied on the sections: PDCD10 (anti-rabbit, 1:200) and Iba1 (anti-goat, 1:200); CXCR2 (anti-rabbit, 1:100) and Iba1 (anti-goat, 1:200). Negative control sections were incubated with nonimmune IgG. Slides were counterstained with DAPI (1:500). The images were acquired using a confocal microscope (LSM800, Carl Zeiss, Jena, Germany).

### Orthotopic Homograft Mouse Model

An orthotopic homograft model was employed by using 6-8 weeks old and mixed sex C57BL/6J mice (20-25 g). Mice were given ox*Pdcd10*- (ox*Pdcd10*) or control-GL261 cells (C) by cerebral orthotopic injection using mouse intracranial stereotactic injection system (RWD, Shenzhen, China). Briefly, mice were anesthetized and immobilized by a head holder. After skin incision, the skull was drilled at the point which located at 1mm anterior and 1.5 mm lateral to the bregma. Then, 2 μl cell suspension containing 3 x 10^5^ cells was slowly injected into the brain by a microsyringe. The needle was slowly retracted and the skin was sutured by a surgical histoacryl. To evaluate whether inhibition of CXCR2 signaling reduced *in vivo* microglia/macrophages recruitments induced by oxPDCD10-GL261 cells, mice were intraperitoneally injected with SB225002 (5 mg/kg, Selleck Chemicals) daily, beginning on the eighth day after implantation for a whole period of 21 days. Twenty-eight days after implantation, the mice were sacrificed. The brain containing homograft tumors were totally removed and prepared for further studies. Iba1-positive cells were quantified using Image J software in high magnification images.

### Statistical Analysis

The statistical analysis was performed using IBM SPSS 23.0 software. Data was described as mean and standard deviation (Means ± SD). Student’s t-test and ANOVA were respectively used for analyzing the differences between two groups and multiple groups. Pearson’s correlation analysis was used to determine the correlation between two groups. Statistical significance was established as *p*<0.05.

## Results

### PDCD10 Overexpression in GBM Patients Was Positively Correlated With Infiltration of GAMs

**Figure 1:**
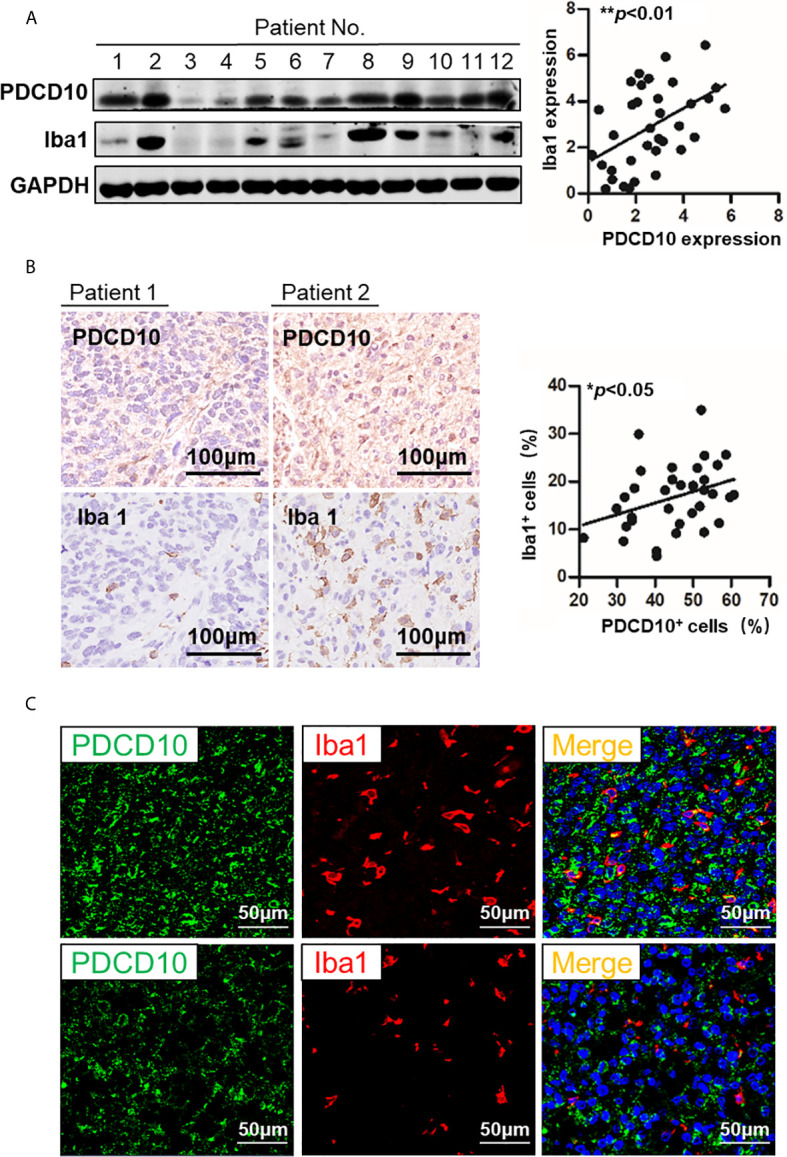
The expression of PDCD10 in glioblastoma (GBM) patients displayed a positive correlation with the infiltration of glioma-associated microglia/macrophages (GAMs). **(A)** Western blot of PDCD10 and Iba1 in GBM patients. Representative blots showed the expression of PDCD10 and Iba1 in GBM patients. Semi-quantification of the blots revealed a positive correlation between PDCD10 and Iba1 expression. **(B)** Immunohistochemical staining of PDCD10 and Iba1 on the adjacent sections of GBM patients. Quantitative analysis revealed that the number of GAMs was positively correlated with that of PDCD10-positive staining cells. **(C)** Immunofluorescent staining of PDCD10 and Iba1 in representative GBM patient. The altered immunoreactivity of PDCD10 (left panel) and Iba-1 (middle panel) were identified in various tumor regions. As shown in double staining (right panel), a massive number of Iba1-labelled GAMs (red) was observed in high PDCD10-immunoreactivity (green) areas. *p< 0.05; **p< 0.01.

The PDCD10 and Iba1 expression in human GBM specimens was detected by Western Blot. The semi-quantification of the blots demonstrated a positive correlation between PDCD10 and Iba1 expression (*p*<0.01) (**Figure 1A**). Moreover, IHC-staining on adjacent slices from different GBM patients showed that Iba1-labelled cells were dominantly observed in those patients with high expression of PDCD10 (**Figure 1B**). Scatter plot analysis indicated a positive correlation between the quantification of PDCD10- and Iba1-positive cells (*p*<0.05) (**Figure 1B**). In addition, the altered expression of PDCD10 in different areas of single slice was also observed by IF-staining (**Figure 1C** left panel). Notably, the Iba1-labelled GAMs were dominantly detected in the area with massive PDCD10 expression (**Figure 1C** middle and right panel).

### Cultured Medium From *PDCD10*-Upregulated GBM Cells Activated Microglia and Macrophages *In Vitro*

**Figure 2:**
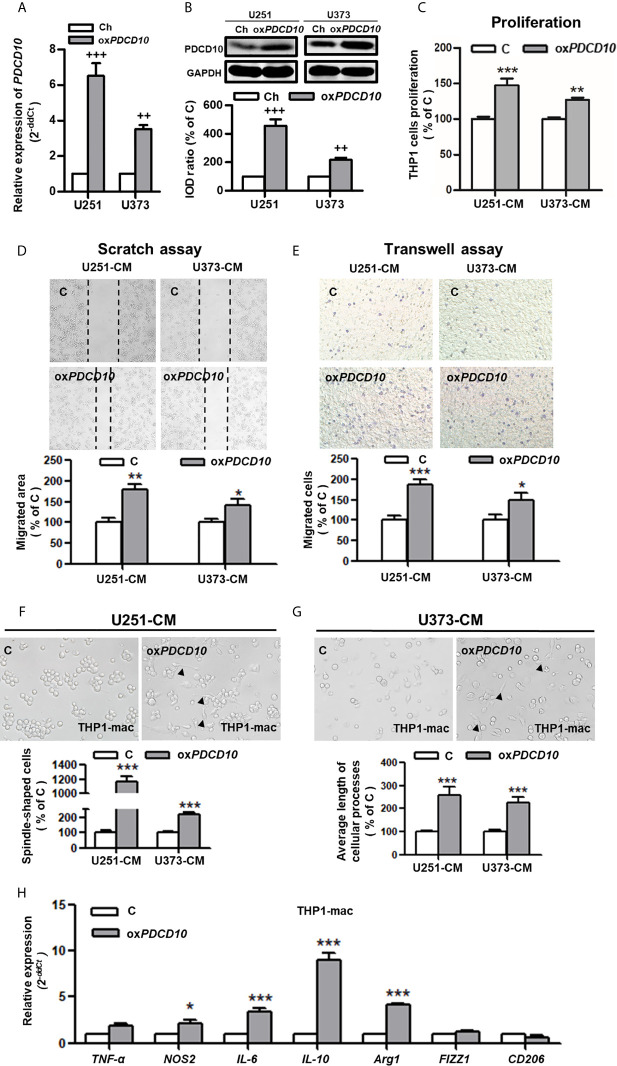
Overexpression of *PDCD10* in human GBM cells activated macrophages *in vitro*. Human GBM cell lines (U251 and U373) were transducted by lentivirus vectors containing human *PDCD10* (ox*PDCD10*) and empty vector (Ch) respectively. **(A, B)** PDCD10 overexpression was confirmed by RT^2^-PCR **(A)** and Western blot **(B)**. Macrophage-like cells (THP1-mac) were generated by the treatment of human THP1 cells with 100 ng/ml prorbol myristate acetate. Then, cells were incubated with conditioned medium (CM) and control medium derived from ox*PDCD10*- (ox*PDCD10*) and control-GBM cells **(C)** respectively. **(C)** Cell proliferation was detected by CCK-8 assay. **(D, E)** Cell migration was measured by scratch assay and transwell assay. For scratch assay **(D)**, migrated area of THP1-mac cells was measured 48 hours after scratching. To perform transwell assay **(E)**, THP1-mac cells were seeded into the insert of transwell system with serum-free medium. CM or control medium was added into the lower chamber. After incubation for 24 hours, the migrated cells were counted after crystal violet staining in a high magnification. **(F, G)** THP1-mac cell morphology study. After incubation with medium derived from ox*PDCD10*-U251 **(F)** or ox*PDCD10*-U373 **(G)** GBM cells, most THP1-mac cells displayed a morphological change including a higher percentage of spindle-shaped cell type as well as a longer cellular process (arrowheads). **(H)** The relative gene expressions in THP1-mac cells were detected by RT^2^-PCR. ^++^p<0.01 and ^+++^p<0.001, compared with Ch; *p<0.05, **p < 0.01 and ***p < 0.001, compared with **(C)**.

To explore the effect of PDCD10 in GBM on microglia and macrophages *in vitro*, two *PDCD10-*overexpressed human GBM cell lines was established (ox*PDCD10*-U251 and ox*PDCD10*-U373) (**Figures 2A, B**). THP1-mac cells cultured with CM derived from ox*PDCD10*-U251 (*p*<0.001) and ox*PDCD10-*U373 (*p*<0.01) showed a significantly higher proliferation rate than that with the corresponding control media (**Figure 2C**). Increase of migration ability in THP1-mac cells mediated by ox*PDCD10*-U251 (*p*<0.01 and *p*<0.001 respectively) and ox*PDCD10*-U373 (*p*<0.05, respectively) were identified by scratch assay (**Figure 2D**) and transwell assay (**Figure 2E**) respectively. After incubation with medium derived from ox*PDCD10*-GBM cells, most THP1-mac cells displayed a morphological change (**Figures 2F, G**), revealing a spindle-shaped cell type as well as a longer cellular process in THP1 cells (*p*<0.001, respectively). Further studies shown in **Figure 2H** revealed that a significant increase in *IL-10, IL-6, arg-1* (*p*<0.001) and *NOS2* (*p*<0.05) was detected in CM-treated THP1-mac cells, indicating a pro-tumorigenic polarization induced by ox*PDCD10*-CM.

**Figure 3:**
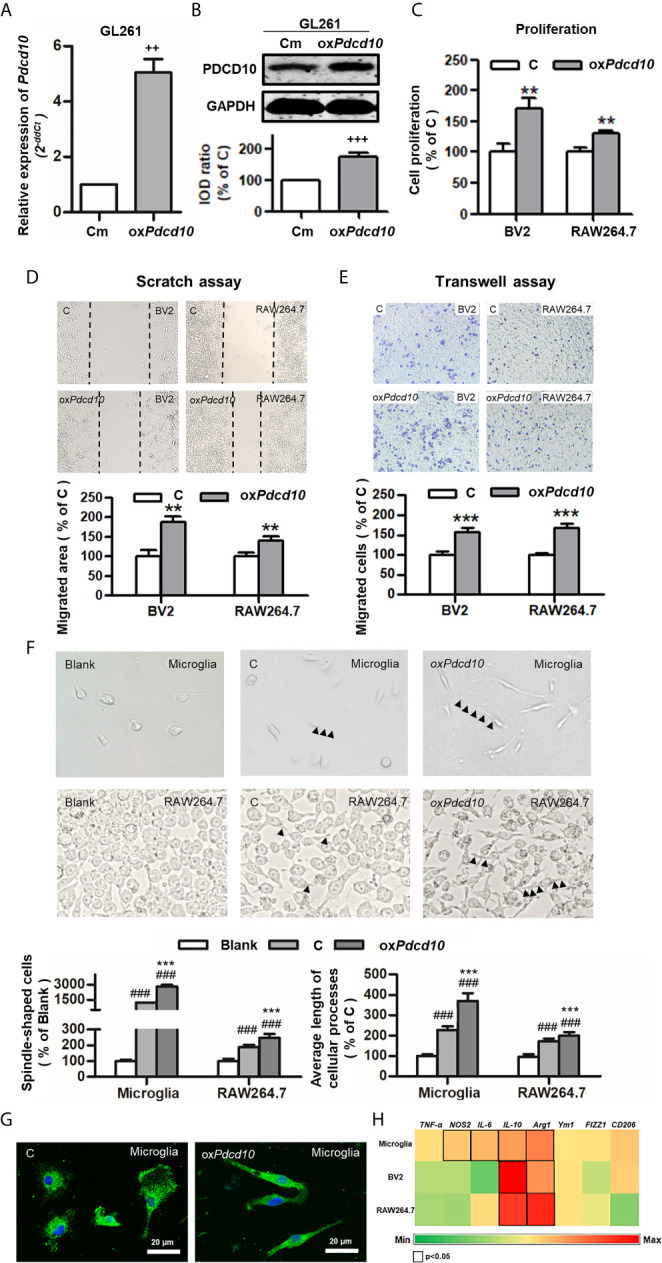
Overexpression of *Pdcd10* in murine GBM cells activated microglia and macrophages *in vitro*. **(A, B)** Stable upregulation of *Pdcd10* in murine GBM cell line GL261 (ox*Pdcd10*) was established by the transduction of lentivirus vector. The control cells (Cm) were transducted with empty vector. The PDCD10 upregulation was confirmed at mRNA **(A)** and protein levers **(B)**. The murine microglia-like cell line (BV2), macrophage-like cell line (RAW264.7) and primary microglia (microglia) were used in the following experiments. **(C)** Medium derived from ox*Pdcd10*-GL261 cells (CM) promoted cell proliferation in BV2 and RAW264.7 cells. Cell proliferation was detected by CCK-8 assay. **(D, E)** CM activated cell migration in BV2 and RAW264.7 cells. For scratch assay **(D)**, BV2 and RAW264.7 cells was seeded in a 6-well-plate followed by media replacement with CM and control medium **(C)** retrospectively. 48 hours after scratching, the migrated area was automatic calculated by image J software. The transwell assay **(E)** was performed using BV2 and RAW264.7 cells. Cells were suspended with serum-free media and seeded into upper insert. CM or control medium was added into the lower chamber. **(F)** The morphological features of primary microglia and RAW264.7 cells. Primary microglia mostly displayed a round-shaped morphology with short cellular processes (left panel). Both CM and control medium induced a spindle-shaped morphologic transformation in both primary microglia and RAW264.7 cells (middle and right panel). In particular, significantly more spindle-shaped cells and longer cellular processes (arrowheads) were identified in both primary microglia and RAW264.7 cells incubated with CM. **(G).** Immunofluorescent staining of Iba1 in microglia showing morphological change induced by CM treatment More spindle-shape microglia were observed in CM-treated group. Scar bar = 20 μm. **(H)** CM induced phenotype polarization in primary microglia, BV2 and RAW264.7 cells. Various gene expressions were detected by RT^2^-PCR and showed in a heat-map (black box indicated statistic difference). ^++^p <0.01 and ^+++^p < 0.001, compared with Cm; **p < 0.01 and ***p < 0.001, compared with C; ^###^p < 0.001, compared with blank.

Subsequently, we performed similar experiments using murine cells. Upregulation of *Pdcd10* was established by lentiviral-*Pdcd10* transduction in murine GBM cell line GL261 (**Figures 3A, B**). A significant increase of cell proliferation was detected in BV2 and RAW264.7 after incubation with medium derived from ox*Pdcd10*-GL261 cells for 72 hours (*p*<0.01, respectively) (**Figure 3C**). Scratch assay reveled a 1.8- and 1.4-fold increase of migrated area in BV2 and RAW264.7 cells treated with ox*Pdcd10*-CM compared with the corresponding controls (*p*<0.01, respectively) (**Figure 3D**). Furthermore, more migrated cells were detected in ox*Pdcd10*-CM treated BV2 and RAW264.7 cells in comparison with control medium treated cells by transwell assay (*p*<0.001, respectively) (**Figure 3E**). To evaluate the morphological and functional change in microglia and macrophages, mouse primary microglia and macrophage-like cells (RAW264.7) were respectively incubated with culture medium (Blank), control GL261-medium (C) and ox*Pdcd10*-GL261 medium (ox*Pdcd10*) for 24 hours. Unstimulated primary microglial and RAW264.7 cells in blank group displayed a round-shape without protrusions (left panel of **Figures 3F, G**). In comparison with blank group, more spindle-shaped cells with longer process were observed in control- and ox*Pdcd10*-groups (*p*<0.001, respectively). In particular, medium generated from ox*Pdcd10*-GL261 induced a more visible morphological change in both primary microglia and RAW264.7 than that in corresponding control groups (*p*<0.001) (**Figure 3F**). IF-staining of Iba1 in primary microglia suggested a similar cell morphological change after treatment by medium from ox*Pdcd10*-GL261 (**Figure 3G**). As shown in **Figure 3H**, a heat-map was used to illustrate the change of the related markers in murine primary microglia, BV2 and RAW264.7 cells. In detail, *IL-10*, *IL-6* and a*rg-1* were significantly elevated after ox*Pdcd10*-CM treatment in comparison with control (*p*<0.05).

### Upregulation of *Pdcd10* in GBM Increased Microglia/Macrophages Recruitment and Promoted Tumor Growth *In Vivo*

**Figure 4:**
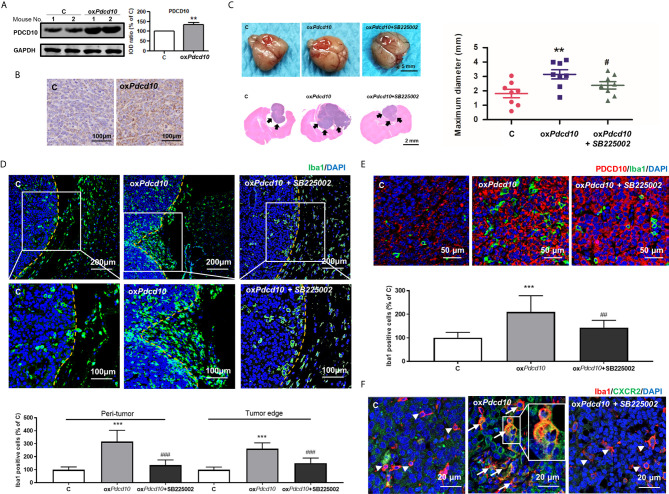
Overexpression of *Pdcd10* in murine GBM cells recruited microglia/macrophages and promoted tumor growth *in vivo*. Mice were cerebral orthotopic implanted with ox*Pdcd10*- (ox*Pdcd10*) or control-GL261 cells **(C)** (n = 8 for each group). The whole brain was removed 28 days after implantation. **(A)** The stable PDCD10 overexpression was confirmed by Western blot. **(B)** Immunohistochemical staining demonstrated PDCD10 overexpression in homograft tumors. Scale bar = 100 μm. **(C)** Overexpression of *Pdcd10* promoted homograft tumor growth, which was reversed by SB225002 treatment. Representative photos showed the homograft tumors (dot-line) in control-, ox*Pdcd10*-mice, and SB225002 treated ox*Pdcd10*-mice. Scale bar = 5 mm. HE-staining demonstrated that homograft tumor (arrows) in ox*Pdcd10* group was larger than that in control group. After treatment with SB225002, the increase of tumor volume was reduced. **(D)** *Pdcd10* overexpression in GL261 cells recruited microglia/macrophages, which was attenuated by SB225002 treatment. Substantial Iba1-positive cells (green) were detected in tumor edge and peri-tumor area (dot-line indicated tumor margin). The lower photos were the enlargement of white box in the upper images respectively. **(E)** Microglia/macrophages infiltration in tumor core induced by *Pdcd10* overexpression was also attenuated by SB225002 application. Double-staining of PDCD10- (red) and Iba1 (green) in tumor core. Scar bar = 50 μm. **(F)** CXCR2 activation in microglia/macrophages mediated by *Pdcd10* upregulation was abolished by SB225002 treatment. Immunoreactivity of CXCR2 (green) was merely detected in cells without Iba1-staining (red, arrowheads) in control mice. Co-labelling of Iba1 and CXCR2 (orange, arrows) was significantly detected in ox*Pdcd10*-tumors. The box (middle photo) was magnified to show the co-localization of CXCR2 and Iba1. Scar bar = 20 μm. **p<0.01 and ***p<0.001, compared with C, ^#^p < 0.05, ^##^p < 0.01 and ^###^p < 0.001, compared with ox*Pdcd10*.

GL261 (C) and ox*Pdcd10*-GL261 cells (ox*Pdcd10*) were orthotopically implanted into mice brain. Stable overexpression of PDCD10 *in vivo* was confirmed by Western blot (*p*<0.01) (**Figure 4A**), IHC- (**Figure 4B**) and IF-staining of PDCD10 (**Figure 4E**). At 28 days after implantation, the maximum diameter of tumor mass was larger in ox*Pdcd10*-mice than that in control mice (*p*<0.01) (**Figure 4C**). To evaluate the microglia/macrophage recruitment, two sectional areas of interest including tumor edge (**Figure 4D**) and tumor core (**Figure 4E**) were examined. Of note, IF-staining of Iba1 (green) revealed a significantly more microglia/macrophage infiltration in peri-tumor area and tumor edge (*p*<0.001, respectively) (**Figure 4D**) in ox*Pdcd10*-group, suggesting a potential chemotaxis effect mediated by PDCD10 on microglia/macrophages which migrated from normal brain tissue towards homograft tumors. As shown in **Figure 4E**, the number of infiltrated microglia/macrophages in tumor core was larger in ox*Pdcd10*-group than that in control group (*p*<0.001), and a positive correlation was revealed between PDCD10 expression and infiltration of microglia/macrophages, which was consistent with our findings in GBM patients. Intriguingly, the aggressive tumor growth and increase of microglia/macrophage recruitments induced by *Pdcd10* upregulation were reversed by the treatment with SB225002 (**Figures 4C–E**).

### Upregulation of *PDCD10* in GBM Cells Promoted CXCL2 Release, Which in Turn Activated CXCR2 Signaling in Microglia and Macrophages

**Figure 5:**
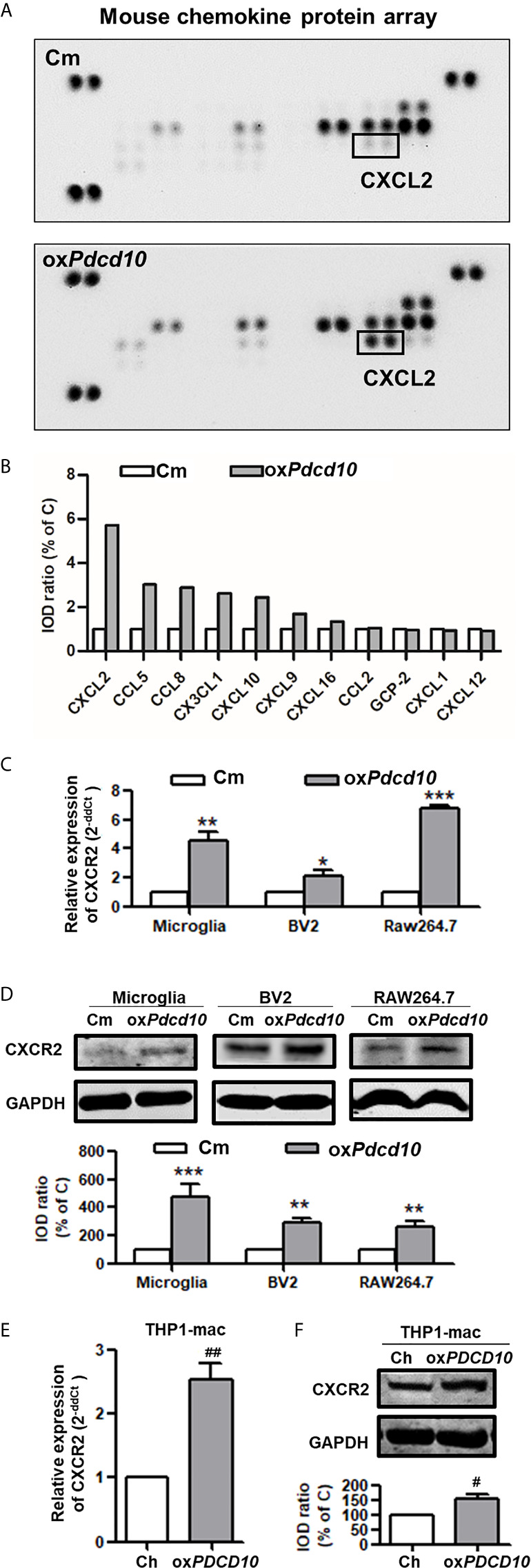
Upregulation of *Pdcd10* in murine GBM cells triggered the release of CXCL2 that in turn activated CXCR2 on microglia and macrophages. **(A)** Different expression of various chemokines in media derived from *Pdcd10*-overexpressed or control-GL261 cells (ox*Pdcd10* or C) was detected by a mouse chemokine array kit. **(B)** Semi-quantification of the dot-blots. CXCL2 was demonstrated as a nearly 6-fold increase in ox*Pdcd10*-medium than that in control medium. **(C, D)** Detection of CXCR2 in murine BV2, RAW264.7 and primary microglia. **(C)** RT^2^-PCR and **(D)** Western blot demonstrated that CXCR2 was upregulated in murine primary microglia, BV2 and RAW264.7 in ox*Pdcd10*-group. **(E, F)** CXCR2 in human THP1-mac cells was detected at mRNA **(E)** and protein levels **(F)**. *p < 0.05, **p < 0.01 and ***p < 0.001, compared with Cm. ^#^p < 0.05 and ^##^p < 0.01, compared with Ch.

To explore the underlying mechanism of PDCD10 in GBM on microglia and macrophages, a mouse chemokine protein array kit including 25 chemokines was used. The dot-blots reflecting protein expression were shown in **Figure 5A**. After semi-quantification, eleven detected chemokines were listed in **Figure 5B**. Among them we outlined that CXCL2 was the one of greatest upregulated chemokines. Then, we further examined the expression of CXCR2 in primary microglia, microglia- and macrophage-like cells. Notably, a significant upregulation in both mRNA (**Figure 5C**) and protein levels (**Figure 5D**) of CXCR2 were detected in mouse microglia, BV2 and RAW264.7 cells treated with medium from ox*Pdcd10*-GL261 cells. Similarly, CXCR2 expression in human THP1-mac cells was upregulated after incubation with ox*PDCD10*-CM than that with control medium (**Figures 5E, F**). Additionally, double-staining of Iba1 and CXCR2 in sections generated from homograft tumors demonstrated an increased expression of CXCR2 on microglia/macrophages in ox*Pdcd10*-tumors than control (**Figure 4F**), while this effect was reversed by SB225002 application (**Figure 4F**).

### Treatment of SB225002 Suppressed CXCR2 Activation and Cell Migration of Microglia and Macrophages Induced by PDCD10-Upregulated GBM Cells

**Figure 6:**
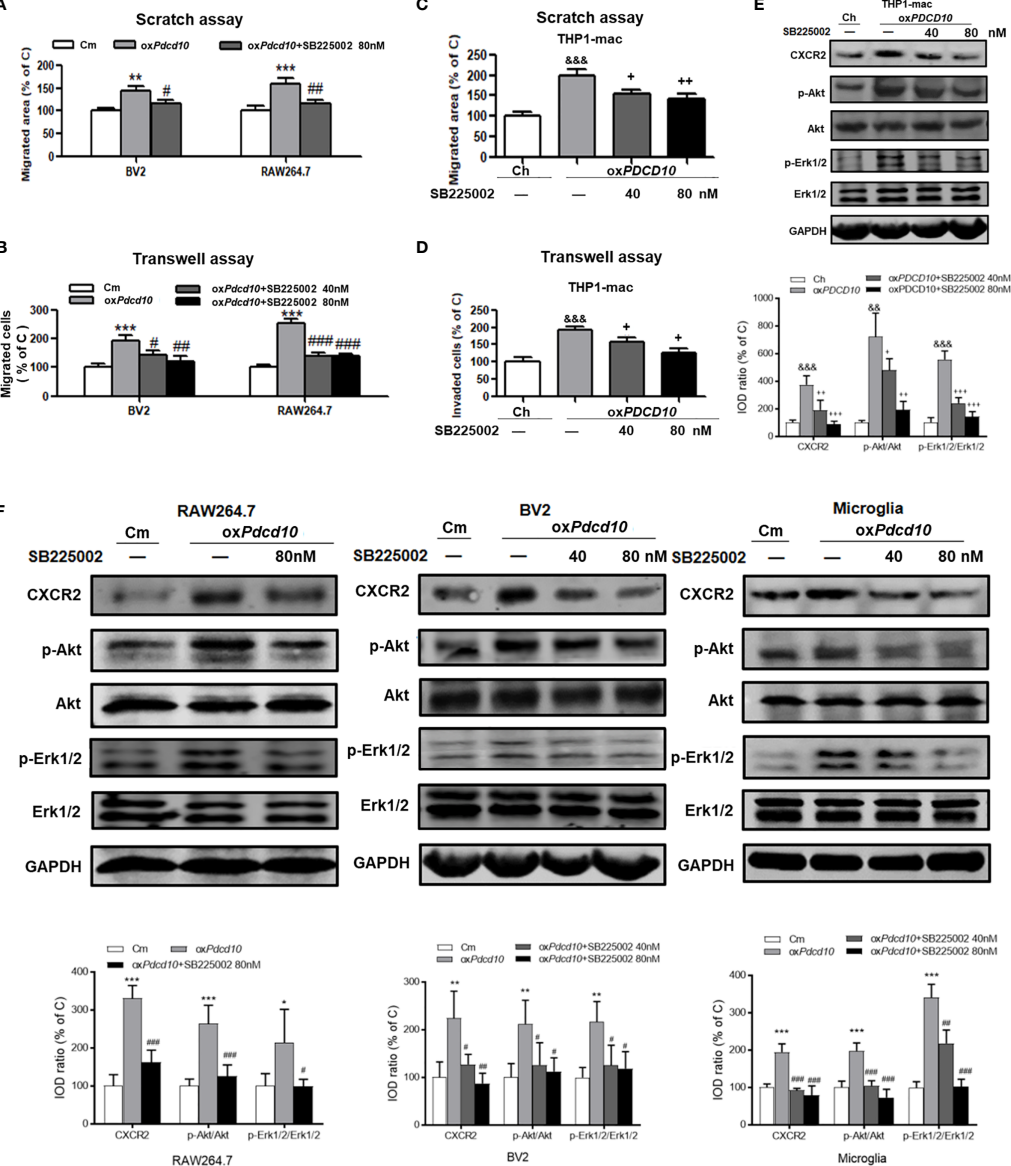
SB225002 treatment suppressed CXCR2 activation and cell migration in microglia and macrophages induced by PDCD10-upregulated GBM cells. Cell migration was detected in murine BV2 and RAW264.7 cells **(A, B)** and in human THP1-mac cells **(C, D)**. For scratch assay **(A, C)**, cells were treated with 40 nM or 80 nM SB225002 for 48 hours. To perform transwell assay **(B, D)**, 40 nM or 80 nM SB225002 was used for cell treatment for 24 hours. CXCR2 and downstream signaling pathways in human THP1-mac **(E)** murine primary microglia, BV2 and RAW264.7 cells **(F)** were detected by Western blot. *p < 0.05, **p < 0.01 and ***p < 0.001, compared with Cm; SB225002 treatment suppressed CXCR2 activation and cell migration in ^#^p < 0.05, ^##^p < 0.01 and ^###^p < 0.001, compared with oxPdcd10; ^&&^p < 0.01, ^&&&^p < 0.001, compared with Ch; ^+^p < 0.05, ^++^p < 0.01 and ^+++^p < 0.001 compared with oxPDCD10.

Murine BV2 and RAW264.7 cells were co-cultured with media from ox*Pdcd10*-GL261 (ox*Pdcd10*) and control GL261 cells (C) respectively. SB225002, a selective CXCR2 antagonist, was applied to BV2 and RAW264.7 cells with optimized concentrations. As a result, the migrated area induced by *oxPdcd10-*CM was reduced by 27% in BV2 cells (*p*<0.05) and 43% in RAW264.7 cells (*p*<0.01) after the administration of 80 nM SB225002 (**Figure 6A**). For transwell assay, both 40 nM and 80 nM of SB225002 were applied. As shown in **Figure 6B**, the number of migrated cells induced by ox*Pdcd10*-CM was significantly reduced by SB225002 treatment (BV2:*p*<0.05 and *p*<0.01, RAW264.7: *p*<0.001 and *p*<0.001, for 40 nM and 80 nM SB225002 application respectively). Additionally, human THP1-mac cells treated with medium from ox*PDCD10*-GBM cells also showed a significant increase of migration ability (*p*<0.001, respectively), which was attenuated by the application of optimized concentration of SB225002 respectively (*p*<0.05, respectively) (**Figures 6C, D**).

Moreover, the signaling pathway was verified by Western blot. As shown in **Figures 6E, F**, the upregulated CXCR2 expression mediated by ox*Pdcd10*/ox*PDCD10*-GBM cells was identified in primary microglia, microglia- and macrophage-like cells respectively, which were abolished upon SB225002 treatment in different concentrations. Following the activation of CXCR2, the phosphorylation of Erk1/2 and Akt were significantly elevated, which was subsequently reversed following the SB225002 application in a dose-dependent manner.

## Discussion

In the past few years, the role of PDCD10 in regulating endothelial angiogenesis and apoptosis were well studied (13). However, there was remarkable controversy regarding the role of PDCD10 in human malignant tumors. Previous reports revealed that PDCD10 in malignant tumors exerted dual functions through a cell-type dependent manner (23, 24). Until now, the role of PDCD10 in GBM is still not clear. A recent publication demonstrates that loss of PDCD10 in GBM promotes cellular behaviors and tumor progression (21). However, the TCGA data raise obvious controversy revealing that *PDCD10* is upregulated in GBM, and associated with poor prognosis (**Figure S1**). PDCD10 triggered a paracrine manner, which in turn affects GBM cells (22), indicating an important role of PDCD10 in pathological TME. It is well known that accumulative evidence demonstrates that TAMs play a pivotal role in regulation of tumor progression. Based on the paracrine mechanism and the direct interaction between tumor cells and TAMs, our present study aimed to explore the potential impact of *PDCD10* in GBM on GAMs and the underlying mechanism. In summary, our study revealed that ①upregulation of PDCD10 is positively correlated with GAMs infiltration in GBM patients; ②upregulation of PDCD10 in GBM cells recruits and activates murine primary microglia, microglia- and macrophage-like cells *in vitro* and promotes tumor growth *in vivo*; ③PDCD10-overexpressed GBM cells increases the release of CXCL2, which activates CXCR2 and downstream Akt and Erk1/2 signaling in primary microglia, microglia- and macrophage-like cells.

GAMs are mainly composed of resident microglia and MDMs. Most previous publications demonstrated that cellular distribution and function of resident microglia and MDMs are similar, and accordingly it was difficult to discriminate them (25, 26). However, recent studies revealed tiny differences in their distributions. MDMs occupy a dominant proportion and are observed in all areas of tumor mass with an extremely high distribution in close proximity to vessels, while resident microglia are often confined to tumor border areas and absent from the tumor core in GBM. These reports also employed flow cytometry or histology to discriminate GAMs by using different markers, such as P2ry12, Tmem119 for microglia and Emilin2, Mertk for MDMs (25, 27, 28). In the present study, we performed indirect co-culture experiments by using various human and murine cells to understand the possible interaction between GBM and microglia and macrophages. Considering the possible differences between residual microglial and MDMs, to avoid the bias, we used macrophage-like (THP1-mac and RAW264.7 from human and murine respectively) and microglia-like cell lines (BV2 from murine) as well as murine primary microglia to confirm our consumption. For instance, the following interaction groups including two human ox*PDCD10*-GBM cell lines (U251 and U373) and THP1-mac, murine ox*Pdcd*-GBM cell line (GL261) and three microglia as well as macrophages (primary microglia, BV2 and RAW264.7), were used to perform multiple cellular behavior studies. Intriguingly, incubation of microglia- and macrophage-like cells with medium derived from *PDCD10*/*Pdcd10*-upregulated GBM cells promoted cell proliferation and migration *in vitro*. The GAMs polarization and the related morphological change were still uncertain. Generally, M0 morphological status of microglia/macrophage displays a round-shape with little protrusions without any stimuli. Amoeboid- and spindle-shape with elongated cellular processes is characterized as M1 or M2 polarization (29–32). GAMs in GBM are usually polarized into M2-phenotype, exerting anti-inflammatory function and promoting tumor progression (33). However, some previous reports indicated that co-cultures with GBM cells resulted in an amoeboid shape change in most microglia cells, which in turn play anti-inflammatory function like M2 macrophage (34, 35). In our study, we demonstrated that ox*PDCD10*-CM induced a larger number of M2-phenotype cells than that induced by control medium. Additionally, a significantly increased transcription of M2-markers, such as *IL-10, IL-6* and *arg-1* was detected in ox*PDCD10*-CM cultured primary microglia, microglia- and macrophage-like cells, suggesting that overexpression of *PDCD10* in GBM triggered a pro-tumorigenic phenotype polarization. Subsequently, we employed an orthotopic homograft mouse model to evaluate the impact of GBM PDCD10 on microglia/macrophages *in vivo*. Compared with subcutaneous tumor model, this model provides similar cerebral microenvironment for tumor growth *in vivo* mimicking pathological status in patients. Moreover, this model also avoided the inflammatory reaction induced by xenograft-tumor, which might influence the evaluation of microglia/macrophages recruitment and activation as well as xenograft tumor growth. As a result, PDCD10 expression in homograft tumor was positively associated with the number of infiltrated microglia/macrophages in both tumor edge and tumor core. Since the cellular function of microglia and MDMs possibly differs, the discrimination and respectively functional analysis of them are urged in our further study. In addition, we found that *oxPdcd10-*mice raised a much bigger tumor mass, suggesting that the recruitment of microglia/macrophages might contribute to GBM growth.

Numerous chemotactic factors in tumor pathological circumstance were identified to function in tumor progression. By binding to corresponding receptors, they activate various types of cells including TAMs, endothelial and tumor cells etc. Previous reports demonstrated that TAMs recruitment and activation by various chemotactic factors are crucial process in tumor progression (12, 36). In the present study, we demonstrated that ox*Pdcd10*-GL261 cells facilitated more than 2-fold release of CCL8, CX3CL1, CXCL10, CCL5 and CXCL2. Of them, CXCL2 was dominantly increased by nearly 6-fold, which attracted our attention to focus on the related signaling pathways. CXCL2 mediates biological functions by interacting with corresponding receptor CXCR2 and activates downstream signaling, such as PI3K/Akt, PLC/PKC, MAPK/p38, ras/Erk1/2 and STAT3 etc (37). CXCR2 is dominantly expressed on TAMs, and plays a role in tumor progression through paracrine manner (10, 38). However, CXCR2 mediated autocrine loop is also demonstrated in tumor pathology. In our study, we found that CXCR2 and its downstream signaling, both Akt and Erk1/2, were activated in both microglia and macrophages after the treatment of ox*PDCD* and ox*Pdcd* GBM medium, resulting in a significant increase of cell migration. The related enhancements could be abolished by specific CXCR2 inhibitor application. Moreover, IF-staining of CXCR2 demonstrated that the activation of CXCR2 was identified not only in ox*Pdcd10*-GBM recruited microglia/macrophages but also in ox*Pdcd10*-GBM cells. More intriguingly, treatment of mice with SB225002 inhibited CXCR2 signaling in both microglia/macrophages and tumor cells (**Figure 4F**). Thus, we assumed that CXCL2-CXCR2 resulted in tumor growth *in vivo* might through both autocrine and paracrine manner.

Taken together, PDCD10 in GBM promotes microglia/macrophages recruitment by increasing cell migration ability, and induces pro-tumorigenic polarization through a paracrine CXCL2-CXCR2 signaling pathway. All these effects finally contribute to an aggressive tumor progression. Thus, our study provides evidence that PDCD10 might be an oncogene which highly involved in GBM pathology.

## Data Availability Statement

The raw data supporting the conclusions of this article will be made available by the authors, without undue reservation.

## Ethics Statement

The studies involving human participants were reviewed and approved by Ethical Committee of Tongji Hospital, Tongji Medical College, Huazhong University of Science and Technology. The patients/participants provided their written informed consent to participate in this study. The animal study was reviewed and approved by Committee on Animal Research of Tongji Medical College, Huazhong University of Science and Technology.

## Author Contributions 

QZ, JW, XY, SW and WT performed the *in vitro*, *in vivo* experiments and Western blot. HF and SZ carried out the immunofluorescent and immunohistochemical staining as well as image acquisition. CY performed PCR analysis. CG and XW were involved in data acquisition. KZ took part in project concept and study design. QZ, JW and KZ participated in statistical analysis and took part in manuscript preparation. HZ, KS and TL took part in critical revision of the manuscript for important intellectual content. KZ, JW, HF and XW obtained funding. All authors contributed to the article and approved the submitted version.

## Funding

This work was supported by National Nature Science Foundation of China under Grant 81602204 (KZ), 81702478(JW) and 81602202 (FH) and by the Scientific Research Starting Foundation for Returned Overseas Chinese Scholars from Tongji hospital, Tongji Medical College, Huazhong University of Science and Technology (2020HGRY009).

## Conflict of Interest

The authors declare that the research was conducted in the absence of any commercial or financial relationships that could be construed as a potential conflict of interest.
