# Supplementary material for: Programmed Cell Death 10 Mediated CXCL2-CXCR2 Signaling in Regulating Tumor-Associated Microglia/Macrophages Recruitment in Glioblastoma
Source: Front Immunol. 2021 May 24;12:637053. doi: 10.3389/fimmu.2021.637053 (PMC8182060; doi:10.3389/fimmu.2021.637053)
Supplement: Supplementary file 1 [file Image_1.pdf]

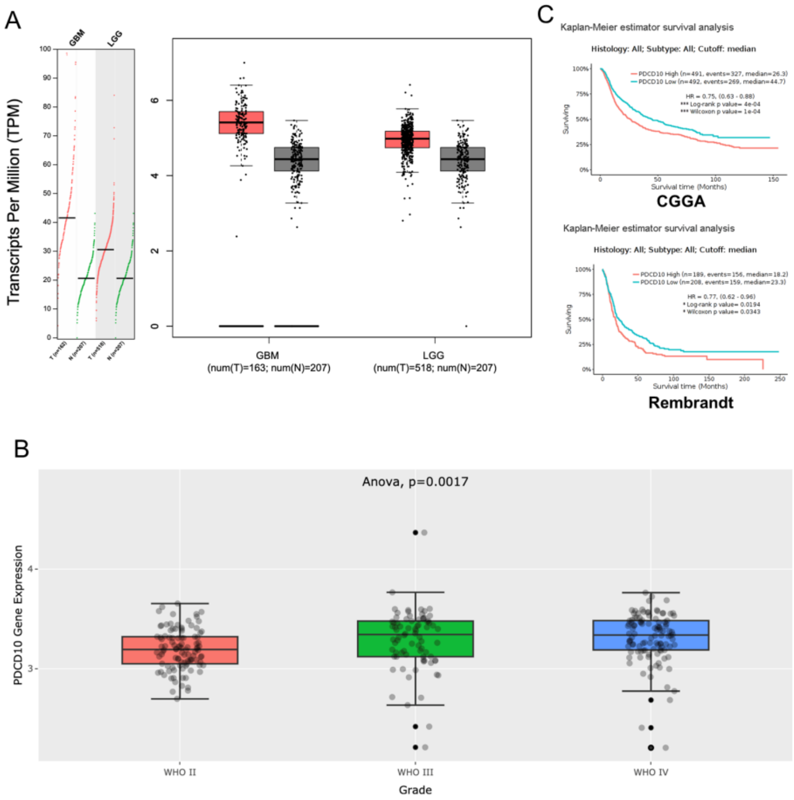

FigureS1.PDCD10 RNA sequencing expression and survival analysis from public databases.

(A) PDCD10 RNA sequencing expression data of GBM(glioblastoma), LGG(low grade glioma) and normal samples from the database of TCGA and the GTEx projects, using a standard processing pipeline on GEPIA(Tang et al. 2017) (http://gepia.cancer-pku.cn/).

(B) PDCD10 gene expression level in different grade of glioma from CGGA (Wang et al. 2015, Liu et al. 2018) (http://www.cgga.org.cn/).

(C) Overall survival analysis from CGGA (Wang et al. 2015, Liu et al. 2018, Bowman et al. 2017) (http://www.cgga.org.cn/) and Rembrandt (http://gliovis.bioinfo.cnio.es/) to verify the prognostic role of PDCD10 in glioma.

Reference:

Bowman, R. L., Q. Wang, A. Carro, R. G. Verhaak, and M. Squatrito. 2017. "GlioVis data portal for visualization and analysis of brain tumor expression datasets." *Neuro Oncol* 19 (1):139-141. doi: 10.1093/neuonc/now247.

Liu, X., Y. Li, Z. Qian, Z. Sun, K. Xu, K. Wang, S. Liu, X. Fan, S. Li, Z. Zhang, T. Jiang, and Y. Wang. 2018. "A radiomic signature as a non-invasive predictor of progression-free survival in patients with lower-grade gliomas." *Neuroimage Clin* 20:1070-1077. doi: 10.1016/j.nicl.2018.10.014.

Tang, Z., C. Li, B. Kang, G. Gao, C. Li, and Z. Zhang. 2017. "GEPIA: a web server for cancer and normal gene expression profiling and interactive analyses." *Nucleic Acids Res* 45 (W1):W98-W102. doi: 10.1093/nar/gkx247.

Wang, Y., T. Qian, G. You, X. Peng, C. Chen, Y. You, K. Yao, C. Wu, J. Ma, Z. Sha, S. Wang, and T. Jiang. 2015. "Localizing seizure-susceptible brain regions associated with low-grade gliomas using voxel-based lesion-symptom mapping." *Neuro Oncol* 17 (2):282-8. doi: 10.1093/neuonc/nou130.
