# Supplementary material for: Programmed Cell Death 10 Mediated CXCL2-CXCR2 Signaling in Regulating Tumor-Associated Microglia/Macrophages Recruitment in Glioblastoma
Source: Front Immunol. 2021 May 24;12:637053. doi: 10.3389/fimmu.2021.637053 (PMC8182060; doi:10.3389/fimmu.2021.637053)
Supplement: Supplementary file 2 [file Image_2.pdf]

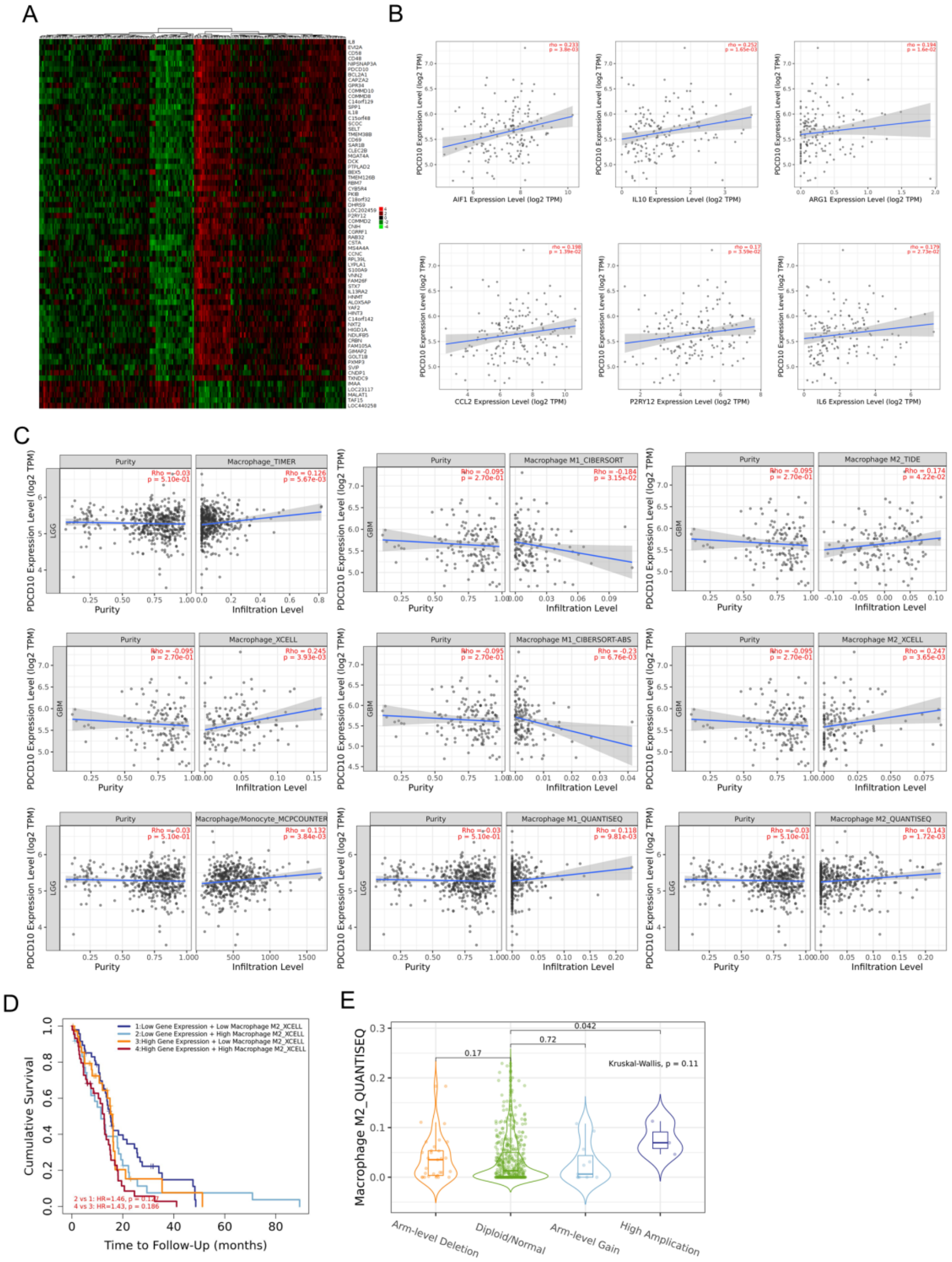

FigureS2. Correlation of PDCD10 with TAMs infiltration level in GBM from TIMER 2.0

(A) Differential analysis of PDCD10 in TCGA_GBM Agilent-4502A dataset shows genes related to TAMs such as CD58, BCL2A1, CAPZA2, GPR34, COMMD10, etc rank top.

(B) PDCD10 expression level is positively correlated to TAMs markers AIL1, IL10, ARG1, CCL2, P2RY12 and IL6.

(C) Macrophages or specifically M1/M2 phenotype macrophages infiltratration estimation value and PDCD10 expression are mostly positively correlated (partial Spearman's correlation was used to remove major confounding factor of tumor purity)
(D) Survival analysis in GBM (n=153) with different level of PDCD10 expression and M2 phenotype macrophages infiltration.
(E) M2 phenotype macrophages infiltration distribution varies among different sCNA status of PDCD10 in LGG TCGA data base by GISTIC2.0 for pairwise comparisons of normal group with each alteration group.
