# Supplementary material for: Programmed Cell Death 10 Mediated CXCL2-CXCR2 Signaling in Regulating Tumor-Associated Microglia/Macrophages Recruitment in Glioblastoma
Source: Front Immunol. 2021 May 24;12:637053. doi: 10.3389/fimmu.2021.637053 (PMC8182060; doi:10.3389/fimmu.2021.637053)
Supplement: Supplementary file 3 [file Table_1.pdf]

Table S1. Primer sequences for RT^2-PCR analysis

Primers	Sequence
**Human**	
PDCD10-Forward	ATGGGAGCTTATCAGCAGTTT
PDCD10-Reverse	TCCATTGACTACTCTGTGTCCA
TNF-α-Forward	TCAGAGGGCCTGTACCTCAT
TNF-α- Reverse	GGAGGTTGACCTTGGTCTGG
NOS2-Forward	AGGTCCAAATCTTGCCTGGG
NOS2-Reverse	ATCTGGAGGGGTAGGCTTGT
IL-6-Forward	ATCAAGACTTACAGGGAGAGGGA
IL-6-Reverse	TGTCAAAGGAGGACCTTGTGG
IL-10-Forward	TCAAGGCGCATGTGAACTCC
IL-10-Reverse	CACGGCCTTGCTCTTGTTT
Arg-1-Forward	GTCTGTGGGAAAAGCAAGCG
Arg-1-Reverse	CACCAGGCTGATTCTTCCGT
FIZZ1-Forward	AGCTCTCGTGTGCTAGTGTC
FIZZ1-Reverse	TGAACATCCCACGAACCACA
CD206-Forward	GACGTGTGCACCTACCTCAA
CD206-Reverse	CCTGGGCTTGACTGACTGTT
CXCR2-Forward	CTAAGTGGCACCTGTCCTGG
CXCR2-Reverse	GGTTGGGTGGTAGTCAGAGC
GAPDH-Forward	TGACTTCAACAGCGACACCCA
GAPDH-Reverse	CACCCTGTTGCTGTAGCCAAA
**Murine**	
PDCD10-Forward	TGGCAGCTGATGATGTAGAAG
PDCD10-Reverse	TCGTGCCTTTTCGTTTAGGT
TNF-α-Forward	CCCTCACACTCAGATCATCTTCT
TNF-α-Reverse	GCTACGACGTGGGCTACAG
NOS2-Forward	GTTCTCAGCCCAACAATACAAGA
NOS2 -Reverse	GTGGACGGGTCGATGTCAC
IL-6-Forward	TAGTCCTTCCTACCCCAATTTCC
IL-6-Reverse	TTGGTCCTTAGCCACTCCTTC
IL-10-Forward	GCTCTTACTGACTGGCATGAG
IL-10-Reverse	CGCAGCTCTAGGAGCATGTG
Arg-1-Forward	CTCCAAGCCAAAGTCCTTAGAG
Arg-1-Reverse	AGGAGCTGTCATTAGGGACATC
FIZZ1-Forward	CCAATCCAGCTAACTATCCCTCC
FIZZ1-Reverse	ACCCAGTAGCAGTCATCCCA
Ym1-Forward	CAGGTCTGGCAATTCTTCTGAA
Ym1-Reverse	GTCTTGCTCATGTGTGTAAGTGA
CD206-Forward	CTCTGTTCAGCTATTGGACGC
CD206-Reverse	CGGAATTTCTGGGATTCAGCTTC
CXCR2-Forward	CAGTTCAACCAGCCCTGACA
CXCR2-Reverse	TACGACGCTGTTTGTGAGCA
GAPDH-Forward	AATGTGTCCGTCGTGGATCTGA
GAPDH-Reverse	GATGCCTGCTTCACCACCTTCT
